# Epithelial membrane protein 3 regulates TGF-β signaling activation in CD44-high glioblastoma

**DOI:** 10.18632/oncotarget.11102

**Published:** 2016-08-05

**Authors:** Fu Jun, Jidong Hong, Qin Liu, Yong Guo, Yiwei Liao, Jianghai Huang, Sailan Wen, Liangfang Shen

**Affiliations:** ^1^ Department of Oncology, Xiangya Hospital, Central South University, Changsha, P. R China; ^2^ Department of Neurosurgery, Xiangya Hospital, Central South University, Changsha, P. R China; ^3^ Department of Pathology, The Second Xiangya Hospital, Central South University, Changsha, P. R China

**Keywords:** gliblastoma, EMP3, TGF-β, TGFBR2, tumorigenesis

## Abstract

Although epithelial membrane protein 3 (EMP3) has been implicated as a candidate tumor suppressor gene for low grade glioma, its biological function in glioblastoma multiforme (GBM) still remains poorly understood. Herein, we showed that EMP3 was highly expressed in CD44-high primary GBMs. Depletion of EMP3 expression suppressed cell proliferation, impaired *in vitro* tumorigenic potential and induced apoptosis in CD44-high GBM cell lines. We also identified TGF-β/Smad2/3 signaling pathway as a potential target of EMP3. EMP3 interacts with TGF-βreceptor type 2 (TGFBR2) upon TGF-βstimulation in GBM cells. Consequently, the EMP3-TGFBR2 interaction regulates TGF-β/Smad2/3 signaling activation and positively impacts on TGF-βstimulated gene expression and cell proliferation in vitro and in vivo. Highly correlated protein expression of EMP3 and TGF-β/Smad2/3 signaling pathway components was also observed in GBM specimens, confirming the clinical relevancy of activated EMP3/TGF-β/Smad2/3 signaling in GBM. In conclusion, our findings revealed that EMP3 might be a potential target for CD44-high GBMs and highlight the essential functions of EMP3 in TGF-β/Smad2/3 signaling activation and tumor progression.

## INTRODUCTION

The epithelial membrane protein 3 (EMP3) is a myelin-related gene that belongs to the peripheral myelin protein 22-kDa (PMP22) gene family [1]. EMP3 is expressed in most tissues, especially in peripheral blood leukocytes, ovary, intestine, and various embryonic tissues, and may regulate cell proliferation, cell-cell interactions, and apoptosis [2–4]. The EMP3 gene has been proposed as a candidate tumor suppressor gene on 19q13.3 in several human solid tumors, such as gliomas, neuroblastoma, pheochromocytoma, non-small cell lung cancer, and esophageal squamous cell carcinoma [5–9]. In these malignancies, EMP3 is frequently inactivated by a hypermethylation-mediated transcriptional gene silencing. In gliomas, hypermethylation in the CpG island of the EMP3 promoter region has been found in 83% and 84% of WHO grades II and III astrocytomas, respectively; in 80% and 73% of WHO grades II and III oligoastrocytomas, respectively; and in 73% and 78% of WHO grades II and III oligodendroglial tumors, respectively [10]. Moreover, constitutive expression of EMP3 in neuroblastoma cell lines induces tumor suppressor-like features in murine xenograft models [11].

Although EMP3 has been implicated as tumor suppressor gene in low grade glioma, its biological function in glioblastoma multiforme (GBM) still remains poorly understood. EMP3 hypermethylation was detected in more than 80% of diffuse, anaplastic astrocytomas and secondary GBMs rather than in primary GBMs [10]. Recently, Mellai et al showed that promoter hypermethylation of the EMP3 gene prevailed in low-grade tumors, especially in gliomas with an oligodendroglial component, and in secondary GBMs upon primary GBMs [6]. Indeed, most primary GBMs lacked EMP3 hypermethylation and frequently over-expressed EMP3 [12]. In addition, higher EMP3 mRNA expression was likely implicated as molecular marker to predict the poor clinical outcome of GBM patients [13]. Collectively, these findings suggested that EMP3 might play an important role in promoting tumorigenesis in primary GBM. However, the molecular mechanisms of EMP3 in primary GBM still await further investigation.

CD44 is a cell-surface marker associated with tumor progression and treatment resistance in glioma. Previous reports indicated that CD44 is a typical biomarker for *Mesenchymal* GBM and predicts unfavorable prognosis in primary GBMs [14, 15]. In this study, we identified EMP3 as a potential tumor-associated gene that is highly expressed in CD44-high primary GBM. Our findings also revealed a crucial role for EMP3 in regulating TGF-β/Smad2/3 signaling activation, which might implicate EMP3 as a potential target for CD44-high GBM.

## RESULTS

### EMP3 expression is highly enriched in CD44-high GBM

**Figure 1:**
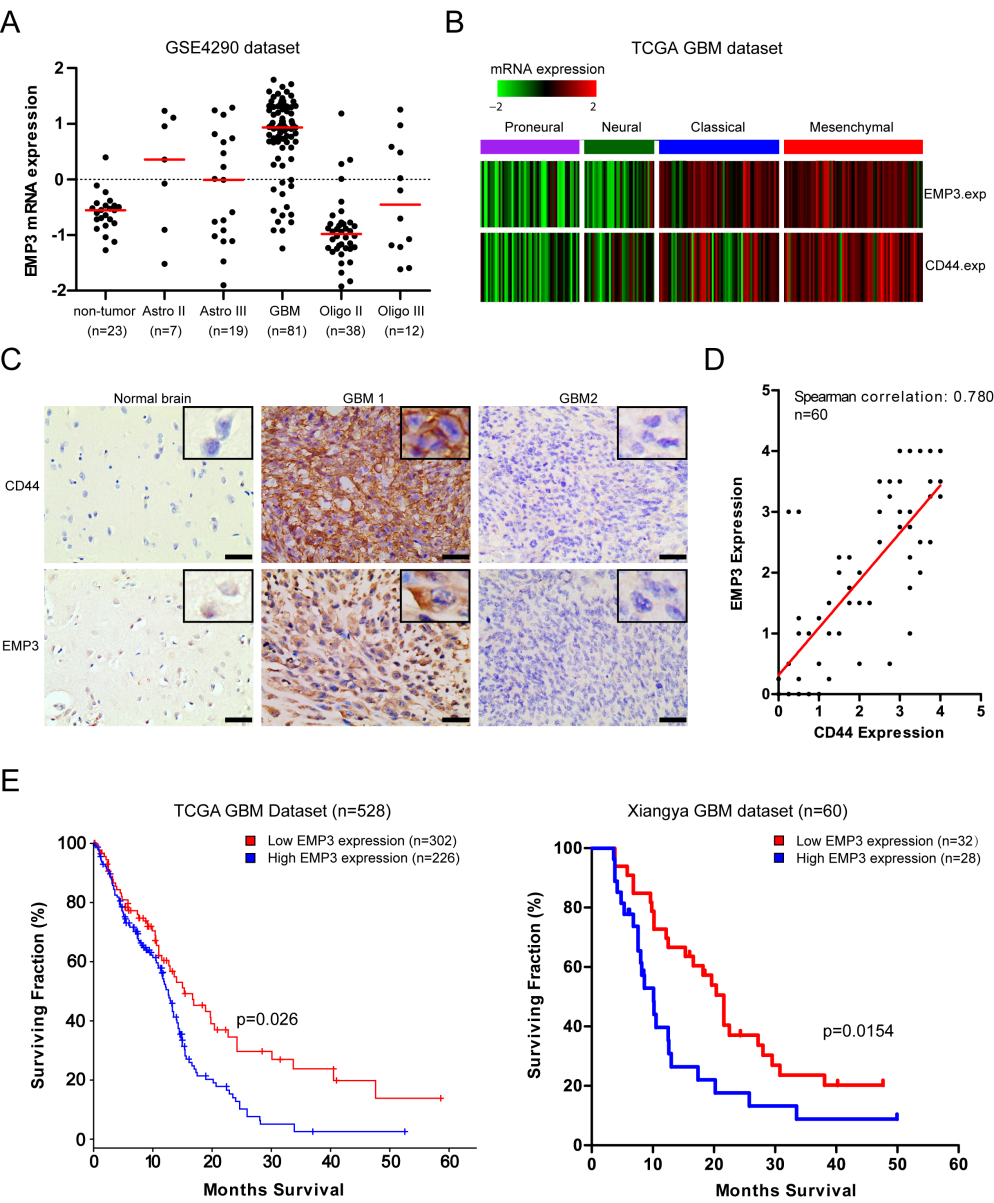
EMP3 is highly expressed in CD44-high GBMs **A**. EMP3 mRNA expression in non-tumor brain tissues and gliomas based on GSE4290 dataset. Red bar indicates the median value of each pathological grade of glioma. **B**. EMP3 expression is highly enriched in TCGA GBM subclasses. **C**. IHC staining of EMP3 and CD44 in human GBM tissues. Bars: 100 μm. **D**. Correlated expression of EMP3 and CD44 in human GBM tissues (Xiangya hospital) (*n* = 60). E. EMP3-high GBMs exhibited unfavorable overall survival time in TCGA GBMs (*n* = 528) and Xiangya GBMs (*n* = 60).

We analyzed mRNA expression of EMP3 in GSE4290 dataset (https://tcga-data.nci.nih.gov/docs/publications/tcga/). Differential EMP3 mRNA expression was observed in gliomas, with the highest expression seen in GBMs (GBMs Vs non-tumor, *P* < 0.0001; GBM Vs grade II or grade III astrocytomas/oligodendrogliomas, *P* < 0.05; Grade II astrocytomas Vs non-tumor, *P* < 0.05. Figure 1A). Consistent with previous reports, oligodendrogliomas (Grade II) exhibited lower EMP3 expression compared to non-tumor, (*P* < 0.05. Figure 1A). Interestingly, TCGA GBM data analysis revealed that EMP3 mRNA expression was mostly distributed in TCGA *Classical* and *Mesenchymal* GBM subtypes compared to those in *Proneural* and *Neural* subtypes, with the highest EMP3 expression observed in *Mesenchymal* GBM subtypes (Figure 1B). Correlated mRNA expression of EMP3 and *Mesenchymal* marker CD44 was observed in TCGA GBM datasets (*n* = 528, Spearman correlation r = 0.605, *P* < 0.0001). We conducted IHC staining on paraffin-embedded archival tumor specimens. No positive staining was observed in isotype IgG control (Supplementary Figure S1A). Differential EMP3 expression was seen in normal brain and GBM tissues (Figure 1C, Supplementary Figure S1B). In addition, we observed the correlated EMP3 and CD44 staining positivity in paraffin-embedded archival tumor specimens (*n* = 60, Spearman correlation r = 0.780, *P* < 0.0001. Figure 1D). Consistent with Ernst et al study [13], survival analysis on both TCGA GBM dataset (*n* = 528) and Xiangya dataset (*n* = 60) revealed that GBM patients with high EMP3 expression exhibited shorter overall survival than EMP3-low patients (Figure 1E). In TCGA GBM dataset, EMP3-high GBM patients showed a median overall survival of 13.2 months (8.6~15.2 months), EMP3-low GBM 16.8 months (9.5~36.5 months, *p* = 0 .026). In Xiangya GBM dataset, overall survival of EMP3-high GBM patients was 10.1 months (5.8~19.2 months) *vs*. 21.6 months (8.1~47.9 months) (*p* = 0 .0154). Therefore, EMP3 might be a tumor-associated gene involved in GBM progression.

### EMP3 depletion attenuates cell proliferation, *in vitro* tumorigenic potential and induces apoptosis in GBM cells

**Figure 2:**
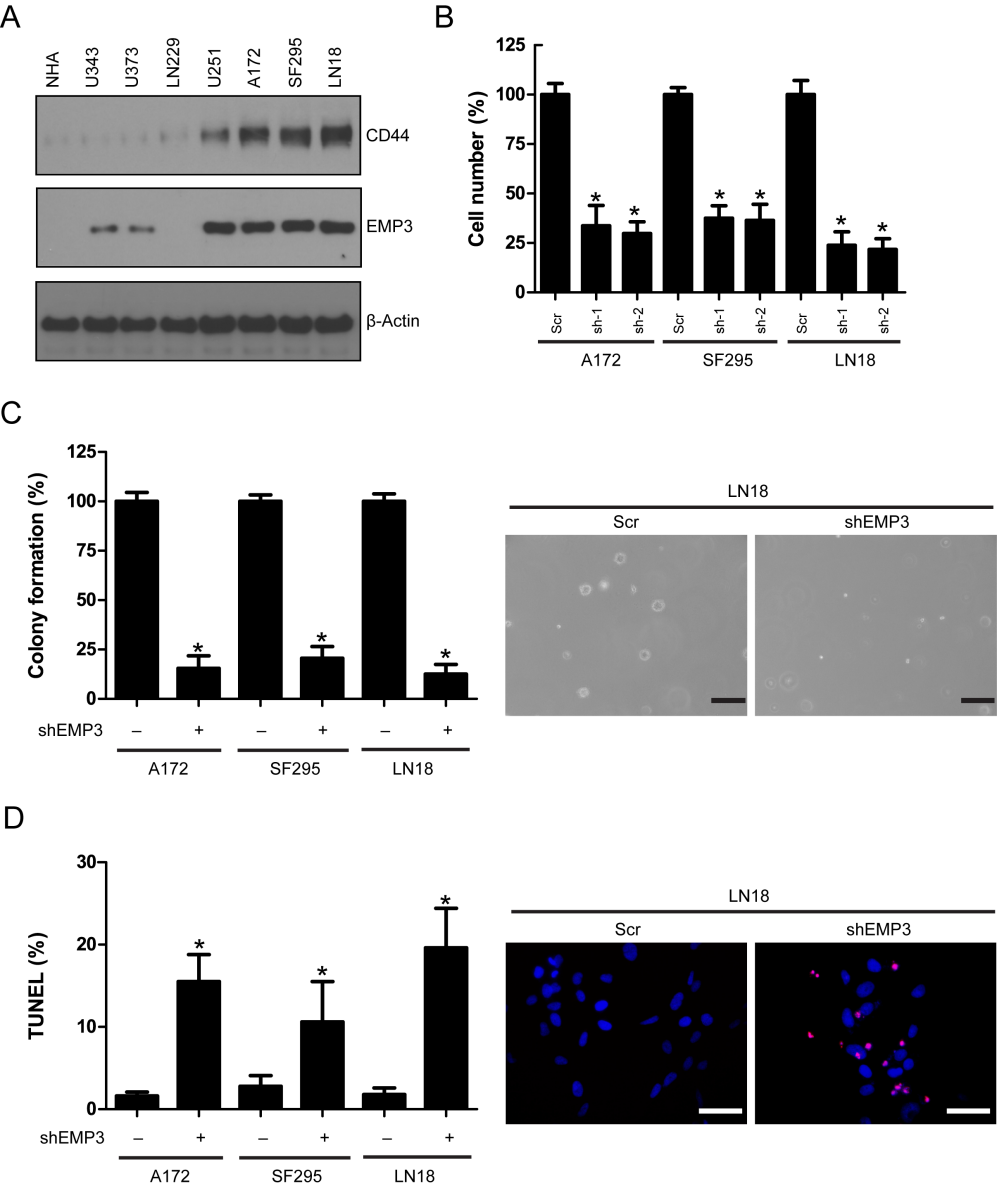
**A**. **Expression of EMP3 and CD44 in human GBM cell lines**. **B**. EMP3 knockdown attenuated glioma cell proliferation in LN18, SF295, and A172 cells. *n* = 4, *P < 0.05, as compared with scramble (Scr) control. **C**. EMP3 knockdown impaired soft agar colony formation in LN18, SF295, and A172 cells. *n* = 4, *P < 0.05, as compared with scramble (Scr) control. Bars: 500 μm. **D**. EMP3 knockdown induced apoptosis in LN18, SF295, and A172 cells. *n* = 4, *P < 0.05, as compared with scramble (Scr) control.

We examined EMP3 protein expression in normal human astrocytes (NHA) and a panel of human GBM cell lines. EMP3 expression was consistent with CD44 status in these cell lines (Figure 2A). To further explore the role of EMP3 in CD44-high GBMs, we depleted EMP3 expression in A172, SF295 and LN18 cells by two specific shRNAs (Supplementary Figure S2). EMP3 depletion by two shRNAs markedly attenuated cell proliferation in all three cell lines, as compared to non-targeting scramble (Scr) control (Figure 2B). Since both EMP3 shRNAs showed similar effect, EMP3 sh-1 shRNA was used for the following experiments. The soft agar assay for colony formation is an anchorage independent growth assay for detecting tumorigenic potential of tumor cells. We showed that EMP3 depletion attenuated the colony formation of three GBM cell lines, suggesting EMP3 might regulate the tumorigenic potential of GBM cells (Figure 2C). Terminal deoxynucleotidyl transferase dUTP nick end labeling (TUNEL) assay showed that EMP3 knockdown increased TUNEL labeling, suggesting EMP3 might exert pro-survival effects on GBM cells (Figure 2D).

Knockdown of EMP3 did not affect cell proliferation in CD44/EMP3-low GBM cell lines U343 and LN229 (Supplementary Figure S3). These results suggested that EMP3 might play a crucial role in CD44-high GBM cells.

### EMP3 interacts with TGFBR2 to regulate TGF-β/Smad2/3 activation in GBM cells

**Figure 3:**
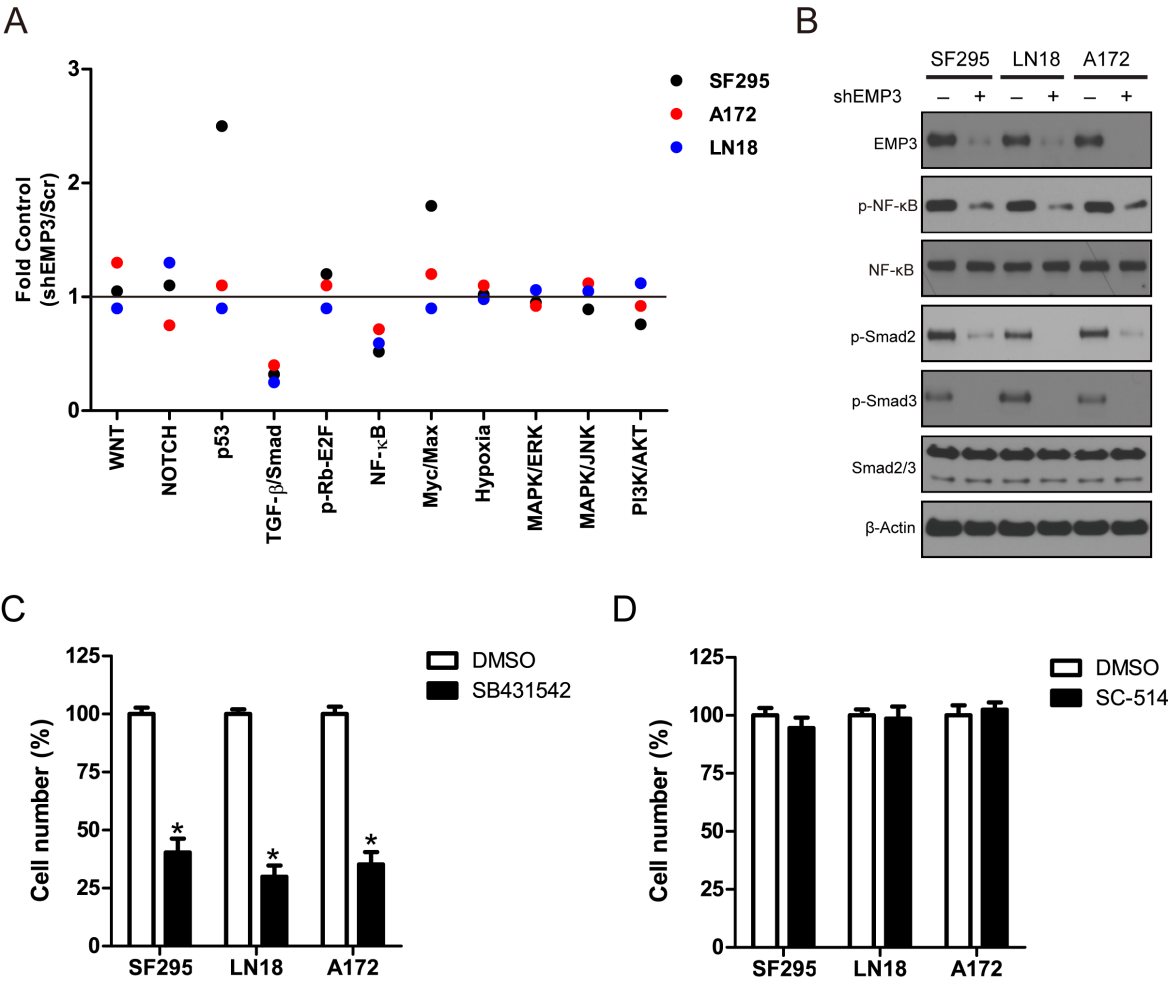
Searching the potential pathway of EMP3 with multi-pathway reporter array **A**. Multiple pathway reporter analysis identified potential cancer-related pathways regulated by EMP3. *n* = 3. **B**. Western blotting validation on candidate pathways following EMP3 depletion in GBM cell lines. **C**. **D**. Inhibitory effect of TGF-β receptor inhibitor SB431542 and IKKβ inhibitor SC-514 on GBM cell lines. GBM cells were seeded in 6-well plates and incubated for 16 hours. SB431542 (10 μM) and SC-514 (2 μM) were added and incubated for 5 days. DMSO was used as solvent control. The cultures were trypsinized, and the number of viable cells in each group was counted with a hemocytometer using 0.2% trypan blue exclusion. *n* = 4, *P < 0.05, as compared with DMSO solvent control.

To explore the molecular mechanisms through which EMP3 regulates GBM cell proliferation and tumorigenesis, a multi-pathway reporter analysis was performed. The result showed that attenuation of EMP3 expression markedly decreased the activities of TGF-β/Smad2/3 and NF-κB in all three GBM cell lines SF295, A172 and LN18 cells (Figure 3A). A strong activation of p53 and Myc/Max signaling was also observed in EMP3-depleted SF295 cells, as compared to Scr control SF295 cells. Consistently, western blotting showed that EMP3 depletion attenuated p-Smad2/3 and p-NF-κB (S536) levels in these GBM cell lines (Figure 3B). To explore the role of TGF-β/Smad2/3 and NF-κB in the regulation of cell proliferation, we treated SF295, A172 and LN18 glioma cells with TGF-β receptor inhibitor SB431542 and IKKβ inhibitor SC-514. These inhibitors specifically blocked TGF-β/Smad2/3 or NF-κB signaling in A172, LN18 and SF295 cells (Supplementary Figure S4). Glioma cell proliferation was greatly suppressed by TGF-β receptor inhibitor SB431542 compared to IKKβ inhibitor SC-514 (Figure 3C, 3D). Moreover, over-expression of constitutively activated IKBα S32A/S36A mutant (protein inhibitor of NF-κB) or siRNA-mediated silencing of Smad2/3 expression also generated effect similar to IKKβ or TGF-β receptor inhibitor in GBM cell lines, respectively (Supplementary Figure S5A, S5B). Therefore, EMP3 might regulate the cell proliferation through modulating TGF-β/Smad2/3 signaling in CD44-high GBM cells.

**Figure 4:**
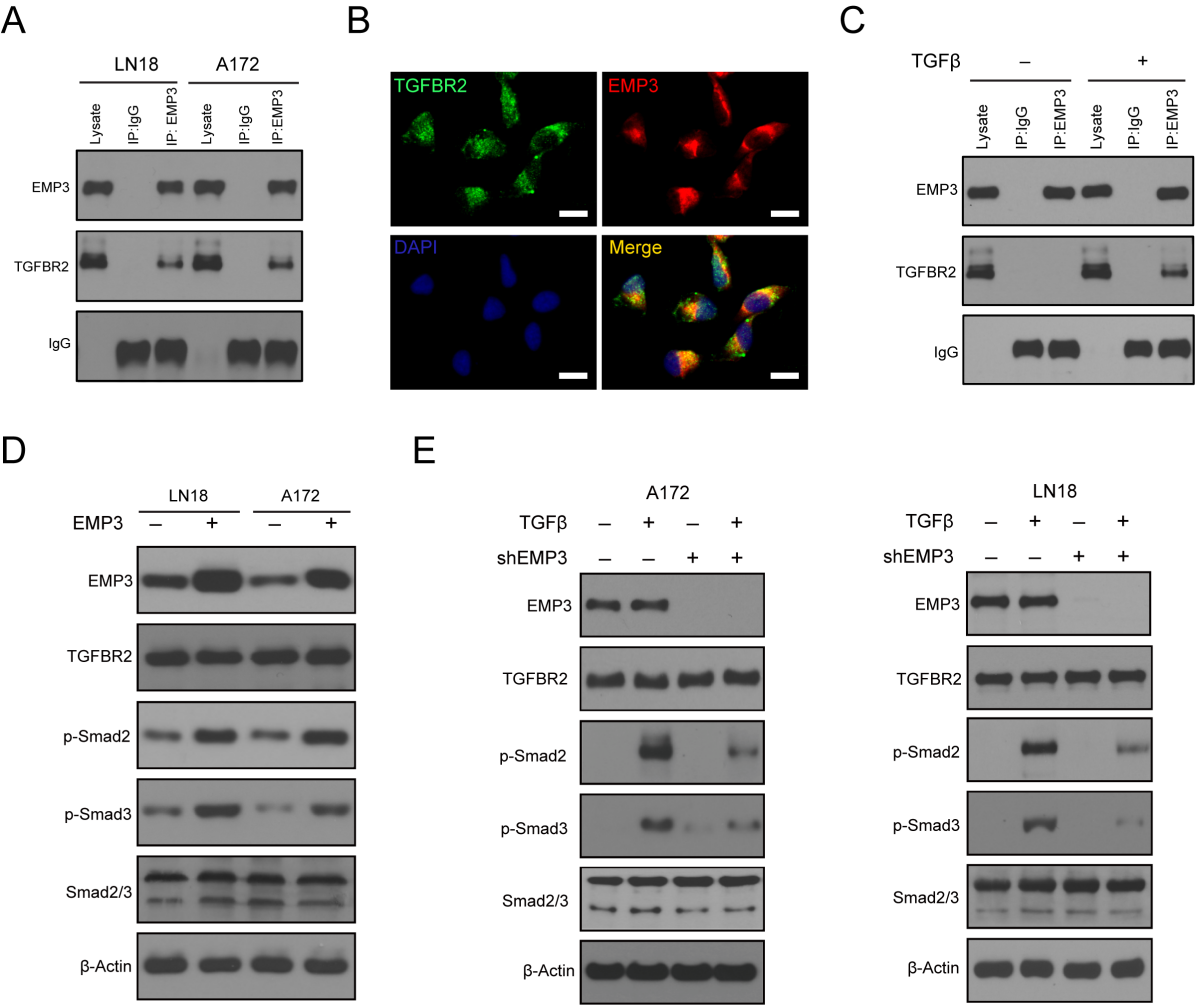
A EMP3 interacts with TGFBR2 in GBM cell lines LN18 and A172. GBM cells were grown in culture media containing 10% Fetal bovine serum. Mouse anti-EMP3 antibody was used to immunoprecipitate TGFBR2 protein. Mouse IgG was used as negative control. **B**. Co-localized staining of EMP3 and TGFBR2 protein in GBM cells. Bars: 10 μm. **C**. TGF-β stimulation induces the interaction between EMP3 and TGFBR2 in GBM cells. After serum deprivation for 36 hours, LN18 cells were incubated with or without TGF-β (5 ng/ml) for 1 hour. Mouse anti-EMP3 antibody was used to immunoprecipitate TGFBR2 proteins. Mouse IgG was used as negative control. **D**. Over-expression of EMP3 promotes Smad2/3 activation in LN18 and A172 cells. GBM cells were grown in culture media containing 10% Fetal bovine serum. **E**. EMP3 depletion attenuates TGF-β-induced TGF-β/Smad2/3 signaling activation in LN18 and A172 cells. After serum deprivation for 36 hours, GBM cells (Scr or shEMP3) were incubated with or without TGF-β (5 ng/ml) for 1 hour. Western blotting was conducted to detect the protein levels of TGF-β/Smad2/3 signaling molecules.

The TGF-β receptor complex is activated through a sequence of events that is initiated by the TGF-β receptor type 2 (TGFBR2) binding to the TGF-β ligand. The TGF-β receptor complex activates the Smad signaling pathway, which includes Smad2, Smad3, and Smad4 to produce the full spectrum of TGF-β responses [16, 17]. Immunoprecipitation analysis showed that EMP3 interacts with TGFBR2 in LN18 and A172 cells (Figure 4A). EMP3 co-localizes with TGFBR2 staining signals in LN18 cells (Figure 4B). The EMP3/TGFBR2 interaction was stimulated by TGF-β in LN18 cells (Figure 4C). Substantial evidence suggests that TGFBR2 regulates the specificity of signaling pathway activation and biological effects of TGF-β [16]. We investigated whether EMP3/TGFBR2 interaction could potentially impact TGF-β/Smad2/3 signaling activation. Over-expression of EMP3 increased p-Smad2/3 levels in LN18 and A172 cells (Figure 4D). After serum starvation for 36 hours, we treated A172 and LN18 cells (Scr or shEMP3) with TGF-β for 10 min. In scramble shRNA control cells, p-Smad2/3 levels were highly up-regulated after treatment with TGF-β (Figure 4E). In contrast, TGF-β treatment of EMP3-depleted cells only resulted in mild increase of p-Smad2/3 compared to that in untreated EMP3-depleted cells (Figure 4E). Thus, EMP3 might be required for TGFBR2 to regulate Smad2/3 activation upon TGF-β stimulation in CD44-high GBM cells.

**Figure 5:**
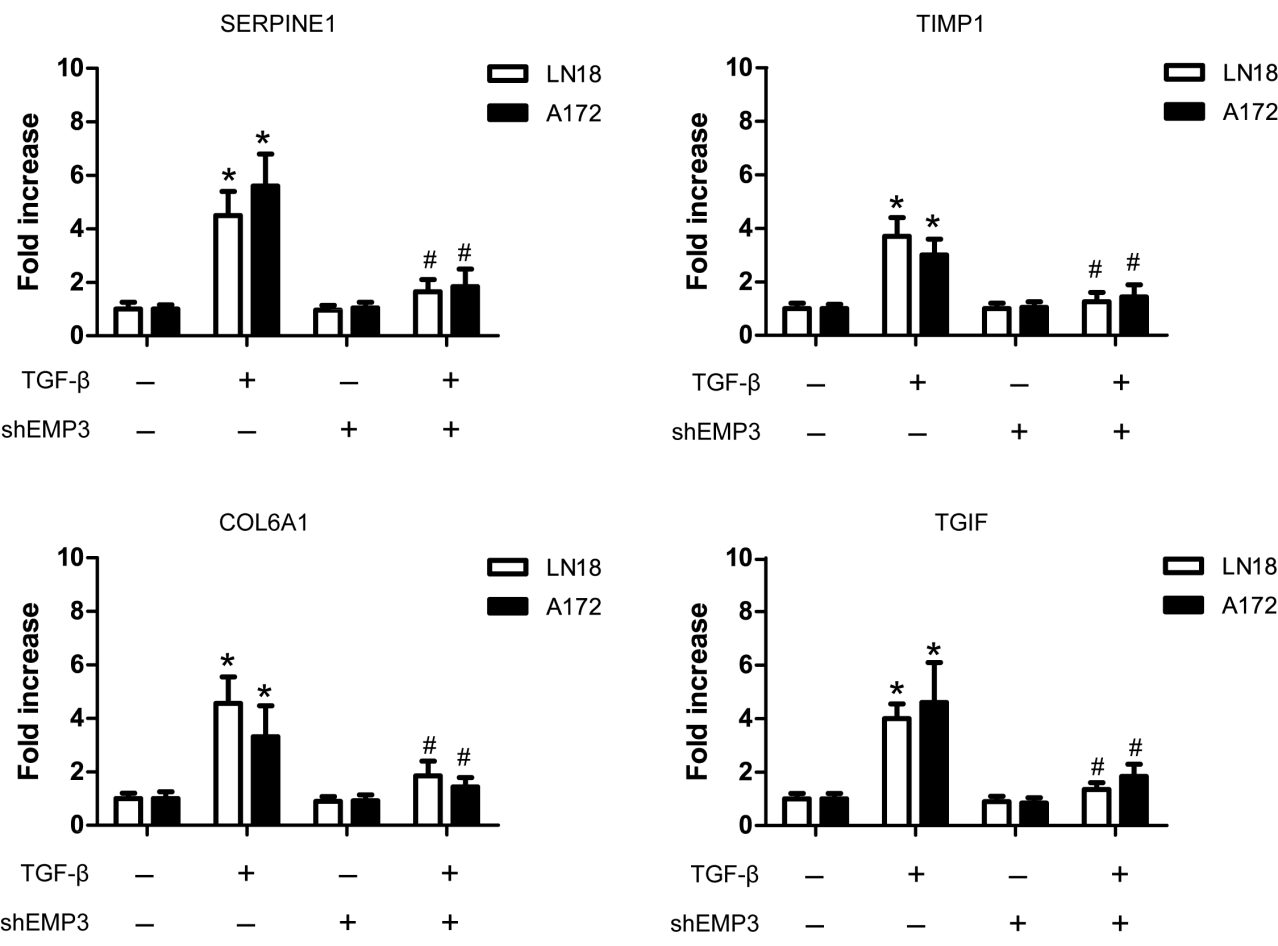
EMP3 regulates TGF-β/Smad responsive gene expression in GBM cell lines. After serum deprivation for 36 hours, LN18 cells (Scr or shEMP3) were incubated with or without TGF-β (5 ng/ml) for 1 hour. RT-PCR was conducted to detect the mRNA levels of TGF-β responsive genes SERPINE1, TIMP1, COL6A1, and TGIF in LN18 and A172 GBM cells following TGF-β stimulation. *n* = 4, *P < 0.05, as compared with scramble (Scr) control without TGF-β stimulation. *n* = 4, ^#^*P* < 0.05, as compared with scramble (Scr) control with TGF-β stimulation.

TGF-β activation in mammalian cells leads to a transcriptional program that typically affects 5-10% of the genes in the genome [18, 19]. High expression of known TGF-β downstream targets, including SERPINE1, TIMP1, COL6A1, and TGIF represented strong TGF-β transcriptional response and contribute to GBM progression [18]. After serum starvation for 36 hours, we treated A172 and LN18 cells (Scr or shEMP3) with TGF-β for 10 min. Real-time PCR analysis showed that TGF-β stimulation increased SERPINE1, TIMP1, COL6A1, and TGIF mRNA expression in EMP3-expressing LN18 and A172 cells (Figure 5). Real-time PCR analysis also revealed that EMP3 depletion led to decreased mRNA expression of these TGF-β responsive genes in TGF-β treated LN18 and A172 cells (Figure 5).

### EMP3 regulates GBM cell proliferation through modulating the activity of TGF-β/Smad2/3 signaling

**Figure 6:**
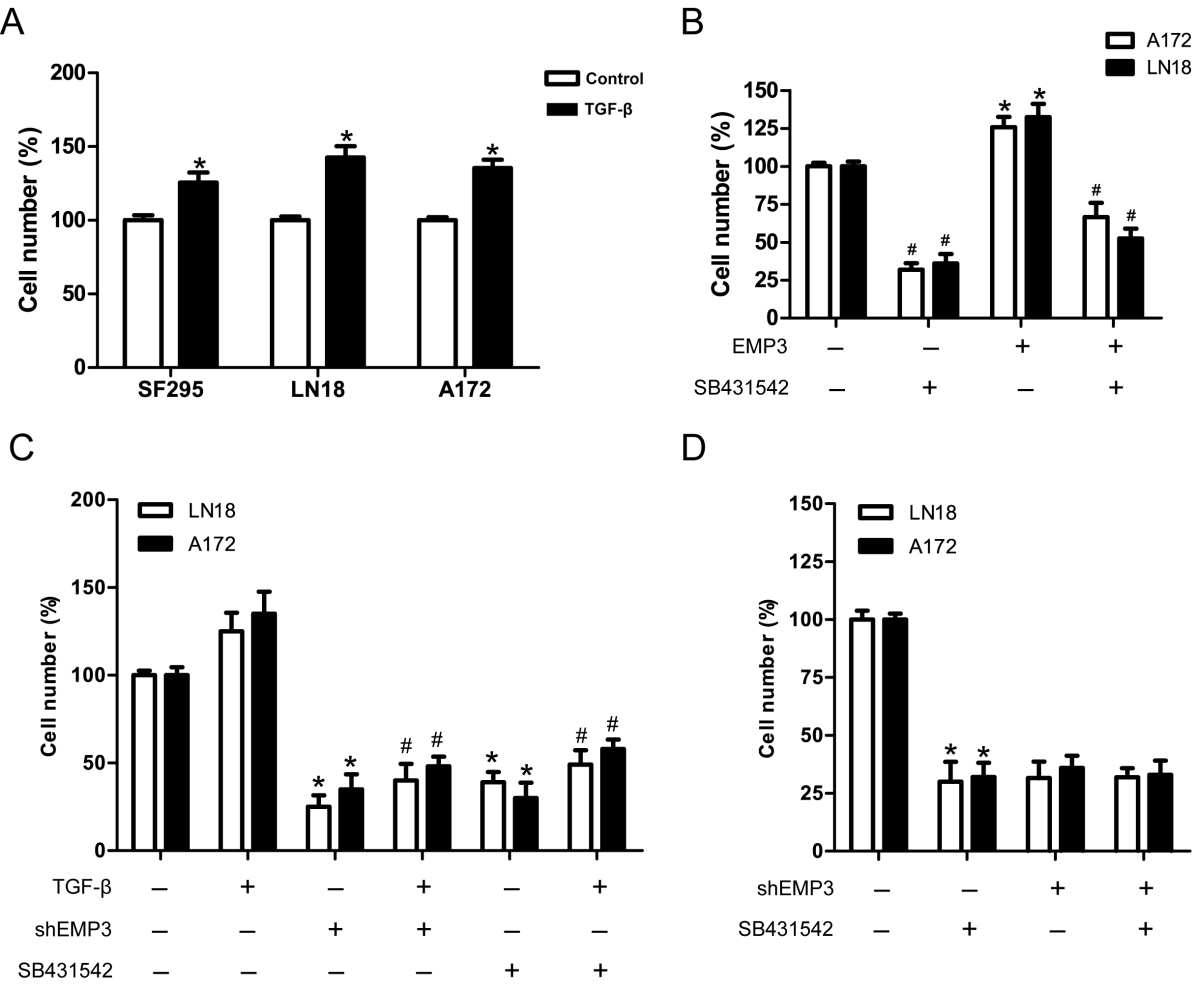
EMP3 is required for TGF-β-driven cell proliferation in GBM cell lines. **A**. TGF-β promotes cell proliferation in GBM cell lines SF295, LN18 and A172 cells. GBM cells were grown in media with or without TGF-β (5 ng/ml) for 5 days. Cell number was quantified using trypan blue exclusion assay. *n* = 4, *P < 0.05, as compared with control without TGF-β treatment. **B**. Over-expression of EMP3 promotes GBM cell proliferation, which was attenuated by SB431542. *n* = 4, *P < 0.05, empty vector control *vs* EMP3 over-expression; *n* = 4, ^#^*P* < 0.05, empty vector control *vs* empty vector control+SB431542, EMP3 over-expression *vs* EMP3 over-expression+SB431542. **C**. GBM cells (Scr or shEMP3) were grown in media with TGF-β (5 ng/ml) or TGF-β receptor inhibitor SB431542 (10 μM) for 5 days. Cell number was quantified using trypan blue exclusion assay. *n* = 4, *P < 0.05, as compared to Scr control without TGF-β. *n* = 4, ^#^*P* < 0.05, as compared to Scr control incubated with TGF-β. **D**. SB431542 effects on EMP3-expressing or EMP3-depleted GBM cells. The number of viable cells in EMP3-expressing without SB431542 treatment was regarded as 100%. *n* = 4, *P < 0.05, as compared with no drug control in EMP3-expressing glioma cells.

TGF-β signaling promotes cell proliferation and tumorigenesis in CD44-high glioma cells, therefore enhancing the capacity of cells to initiate tumors [20, 21]. Our data showed that TGF-β promoted GBM cell proliferation in LN18, SF295 and A172 cells (Figure 6A). Similarly, Over-expression of EMP3 also promoted GBM cell proliferation in A172 and LN18 cells, which was attenuated by TGF-β receptor inhibitor SB431542 (Figure 6B). The effect of TGF-β was markedly abrogated by SB413542 or EMP3 depletion (Figure 6C). In order to confirm that the effect of EMP3 on GBM cell proliferation is mediated by TGF-β signaling, we treated EMP3-expressing or EMP3-depleted GBM cells (LN18, A172) with SB431542. SB431542 markedly inhibited cell proliferation in EMP3-expressing LN18 and A172 cells. In contrast, no strong inhibitory effect of SB431542 on cell proliferation was observed in EMP3-depleted LN18 and A172 cells (Figure 6D). These results suggest that EMP3 might regulate GBM cell proliferation *via* modulation of TGF-β signaling activation.

### EMP3 regulates GBM cell proliferation and tumor progression in murine xenograft models

**Figure 7:**
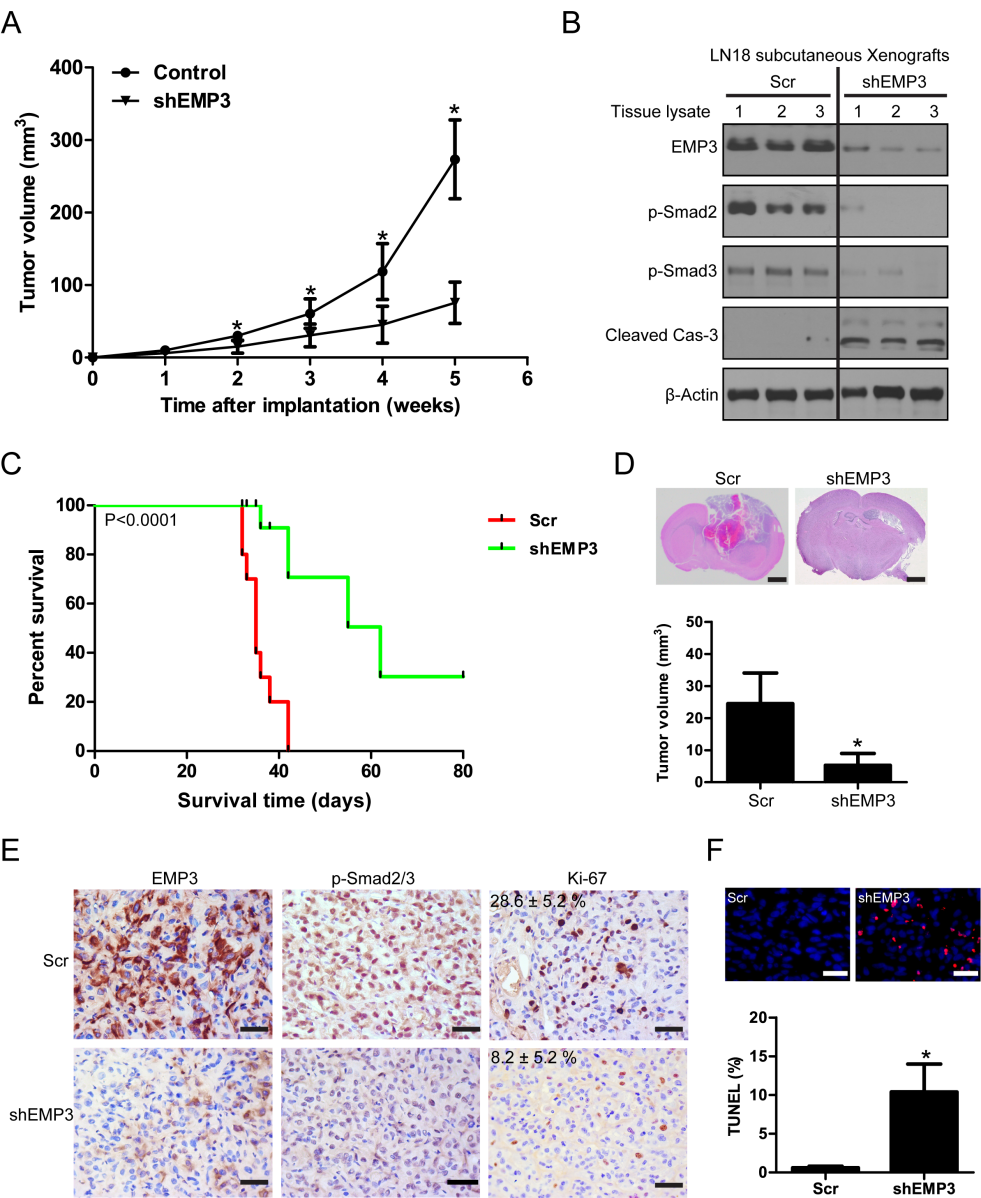
Targeting EMP3 attenuates ***in vivo*** tumor growth and prolongs tumor-bearing mice survival. **A**. EMP3 depletion attenuated subcutaneous tumor growth. *n* = 6, *P < 0.05, as compared with scramble (Scr) control. **B**. Western blotting analysis on protein lysates from subcutaneous tumors with or without EMP3 depletion at week 5 after implantation. **C**. Targeting EMP3 prolongs tumor-bearing mice survival in an orthotopic xenograft model (*n* = 10). **D**. EMP3 depletion attenuates *in vivo* tumor growth in an orthotopic xenograft model. *n* = 6, *P < 0.05, as compared with scramble (Scr) control. Bars: 1 mm. **E**. Immunostaining of the intracranial tumors at day 30 after implantation. The tissue sections were incubated with antibodies against indicated antibodies (EMP3, p-Smad2/3 and Ki-67). Diaminobenzidine was used as a chromogen, followed by counterstaining with hematoxylin. Bar, 50 μm. Ki-67, Scr *versus* shEMP3 (*n* = 6), *P* < 0.05. **F**. EMP3 depletion induces apoptosis in intracranial xenografts. Incidence of apoptosis was determined by TUNEL staining. Bar, 50 μm. *P < 0.05, Scr *versus* shEMP3.

The *in vitro* findings that targeting EMP3 suppressed cell proliferation, impaired tumorigenesis, and induced apoptosis in CD44-high GBM cell lines were carried over to observations with *in vivo* studies. The effect of EMP3 depletion was examined in mice with LN18 glioblastoma subcutaneous xenografts. The group of mice implanted with LN18/Scr glioma cells developed tumors more rapidly than those with LN18/shEMP3 cells. The tumor volume in shEMP3 groups was significantly smaller than the Scr group (*P* < 0.05) (Figure 7A). Western blotting analysis showed that EMP3 depletion attenuated p-Smad2/3 expression and induced caspase-3 activation in xenograft lysates (Figure 7B). We next evaluated the effect of EMP3 depletion in an intracranial xenograft model. The animals were intracranially inoculated with LN18 glioma cells with or without EMP3 knockdown (*n* = 16). The primary endpoint was to evaluate animal survival (*n* = 10). The median duration of survival of the LN18/Scr group was 35 days. The median duration of survival for animals in the LN18/shEMP3 group was extended to 62 days (Scr *vs* shEMP3, *P* < 0.0001) (Figure 7C). In addition, we also examined the *in vivo* effect of EMP3 depletion on TGF-β/Smad2/3 signaling activation. Mice from each group (*n* = 6) were euthanized 30 days after intracranial inoculation. H&E staining on mice brain sections revealed apparent intratumoral necrosis and larger tumor bulk in the LN18/Scr group, as compared with the LN18/shEMP3 group (Figure 7D). Xenografts in the LN18/shEMP3 group showed decreased EMP3, p-Smad2/3, and Ki-67 expression, as compared with the LN18/Scr control group (Figure 7E). TUNEL analyses identified an elevated percentage of apoptotic cells in the EMP3-depleted tumors (Figure 7F). Therefore, EMP3 might serve as a potential therapeutic target for combating CD44-high GBM.

### EMP3 expression correlates with TGF-β/Smad2/3 signaling activation in human GBM tissues

**Figure 8:**
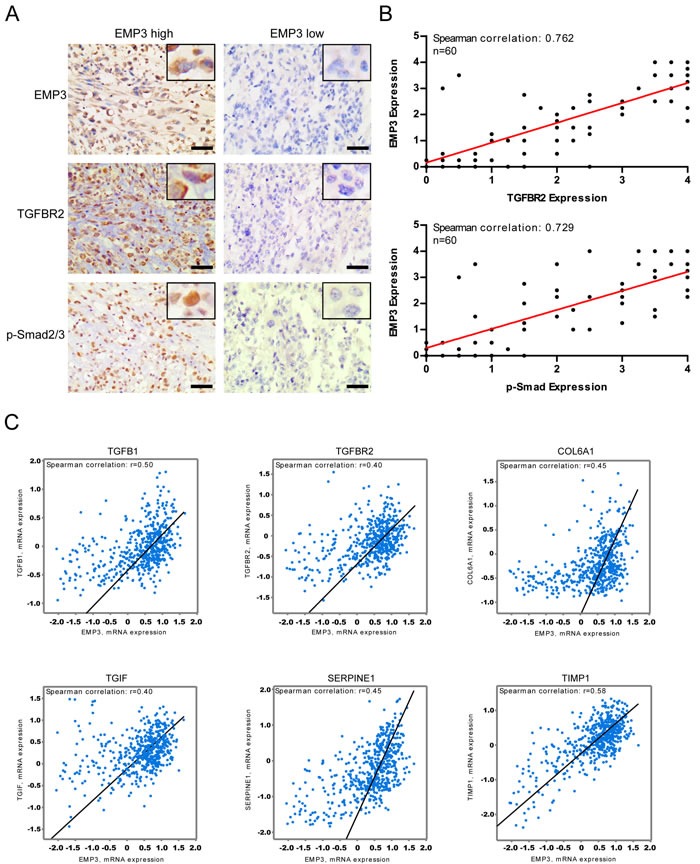
**A**. Immunohistochemical staining of EMP3, p-Smad2/3, and TGFBR2 in primary GBM samples; Bars: 100 μm. **B**. EMP3 protein expression is correlated with TGFBR2 or p-Smad2/3 in GBM samples (*n* = 60). **C**. EMP3 mRNA expression is consistent with TGF-β/Smad2/3 pathway activation in TCGA GBM samples (*n* = 528).

Given the strong effect of EMP3 knockdown on CD44-high GBM cells, we set out to explore the correlation between EMP3 expression levels and TGF-β/Smad signaling activities in a panel of GBM specimens by immunohistochemistry (*n* = 60). Intriguingly, there was a remarkable positive correlation between EMP3 expression and TGF-β/Smad2/3 signaling activation in GBMs. Indeed, EMP3 expression was significantly higher in those GBMs that showed high TGFBR2 or p-Smad2/3 expression (Figure 8A, 8B). Overall, tumors with very low EMP3 expression were mostly negative for TGFBR2 or p-Smad2/3 staining (Figure 8A, 8B). Additionally, TCGA dataset analysis confirmed that most of the samples with high EMP3 mRNA expression also exhibited strong expression in TGF-β, TGFBR2 and TGF-β responsive genes including SERPINE1, TIMP1, COL6A1, and TGIF (*n* = 528, Figure 8C). Altogether, these data might suggest that EMP3 function is especially relevant for the TGF-β/Smad2/3 signaling activation in CD44-high GBMs. In conclusion, these findings might propose EMP3 as an important oncogene in primary GBMs and highlight the essential functions of EMP3 in TGF-β-stimulated Smad2/3 activation and tumorigenesis.

## DISCUSSION

Glioblastoma (GBM) is the most common malignant primary central nervous system (CNS) tumor in adults and remains resistant to current therapies [22]. Ample evidence exists to argue that GBM, as defined by histopathologic criteria, actually represents multiple distinct molecular entities [23]. GBM can be segregated into distinct molecular subclasses based on gene expression signatures and genomic abnormalities. While the precise classifications have varied in the literature two subtypes, termed *Proneural* and *Mesenchymal*, appear robust and generally consistent among the classification schemes [23–25]. GBMs in the *Mesenchymal* subclass are predominantly primary tumors that arise de novo, and, in some studies, exhibit a worse prognosis compared to *Proneural* subclass [15, 26, 27, 28, 29]. CD44 is a typical biomarker for *Mesenchymal* GBM and predicts unfavorable prognosis in primary GBMs [14, 15]. Anido et al revealed the crucial role of CD44-high glioma stem cells in tumor initiation and progression [21]. Therefore, further understanding the regulatory mechanisms in CD44-high GBM cells may help to develop therapeutic strategies in the future.

EMP3, was previously reported to be a tumor suppressor gene for several solid tumors, and is drawing attention as a novel prognostic marker, since its expression level or hypermethylation of the promoter region is associated with clinical prognosis in these cancer types [5]. However, recent findings suggested that the function of EMP3 in human cancers seems to be multi-facet. EMP3 over-expression promoted cancer cell proliferation and migration through activating ErbB2-PI3K-AKT pathway in patients with upper urinary tract urothelial carcinoma [30]. EMP3 was up-regulated in primary breast carcinoma tissues and regulated cell proliferation and invasion in SK-BR-3 cells [31]. Hsieh et al revealed the the tumor progressive effects of EMP3 through PI3K/Akt pathway and uPA/MMP-9 cascade in hepatocellular carcinoma cells [32]. In glioma, the function of EMP3 as a tumor suppressor gene still remains to be controversial. Li et al indicated that EMP3 over-expression is involved in oligodendroglial gliomas retaining chromosomes 1p and 19q and does not support EMP3 as the target tumor suppressor gene on chromosome 19q13 in oligodendroglial gliomas [33]. Several other studies also noticed the over-expression of EMP3 in primary GBMs and predicted poor clinical outcome [12, 13]. In line with these findings, we observed the highest expression of EMP3 in primary GBMs compared to non-tumor brain tissues and lower grade gliomas. Importantly, We found that EMP3 is highly expressed in CD44-high GBM cells; Depletion of EMP3 expression suppressed cell proliferation, impaired tumorigenic potential, and induced apoptosis in these GBM cells. Therefore, EMP3 might be a potential regulator and potential target in CD44-high GBM. Further, the differential function of EMP3 in glioma (as oncogene or tumor suppressor gene) might depend on the pathological grades (low grade glioma or GBM), genetic background (1p/19q LOH or 1p/19q retained) or glioma subtypes.

The function of EMP3 has been proposed to be related to membrane receptors [34]. Cooperation among EMP3 and these membrane receptors may allow integrated intracellular signaling that modulates tumor growth and invasion. EMP3 can interact with membrane receptor P2×7 to regulate apoptotic process following P2×7 receptor activation [34]. The functional crosstalk between ErbB2 and EMP3 activated the ErbB2-PI3K-AKT pathway to promote cancer cell proliferation and migration in urothelial carcinoma [30]. Because EMP3 are widely expressed, even in cells lacking P2×7, this would suggest it may have additional integral membrane protein partners that link EMP3 proteins to various signaling pathways [34]. Identification of such proteins will be an important goal for the future together with understanding the mechanisms involved in tumorigenesis when EMP3 proteins are over-expressed. In this study, we identified TGFBR2 as a potential interacting partner of EMP3 in CD44-high GBM cells. The EMP3-TGFBR2 interaction regulates TGF-β/Smad2/3 signaling activation and positively impacts on TGF-β-stimulated gene expression and cell proliferation in CD44-high GBM cells. The contrary function that EMP3 exerts in glioma might reflect the differential requirement for EMP3/TGF-β signaling to regulate tumor progression in low grade glioma or primary GBM. We also noticed the effect of EMP3 on NF-κB activity, suggesting EMP3 might exert some unknown functions *via* NF-κB signaling in GBM cells. Nevertheless, the differential activation of p53 or Myc/Max following EMP3 knockdown in a certain GBM cell lines might result from the distinct genetic characteristics or signaling network in these cell lines. Collectively, our findings might provide novel insight into the regulatory mechanism of EMP3 in GBM.

Tumor cells often use TGF-β signaling to increase epithelial-to-mesenchymal transition, invasion, and metastasis [35–39]. Although TGF-β signaling suppresses proliferation of certain carcinoma cells and is well known to be a tumor suppressor, it promotes proliferation of tumors of non-epithelial origin [40–43]. In addition to induction of proliferation, the TGF-β pathway has also been implicated in invasion, tumor growth, and intratumoral angiogenesis of glioma [44–47]. These multiple roles of TGF-β in glioma progression have promoted the development of therapeutic agents based on the inhibition of the TGF-β pathway [48, 49]. The inhibition of the TGF-β pathway impaired the maintenance of CD44-high glioma stem cell population and inhibited the capacity of cells to initiate tumors [21]. In the present study, the finding that EMP3 regulates the cell proliferation and tumorigenesis of CD44-high GBM cells sparked our interest in testing the effect of EMP3 depletion on *in vivo* GBM models. Our results indicated that EMP3 depletion suppressed tumor growth and induced apoptosis in murine xenograft models. The benefits of targeting EMP3 were confirmed *in vivo*, with significantly improved survival time seen in the EMP3 knockdown group compared to that in the control group. Attenuation of TGF-β/Smad2/3 activity and induction of apoptosis can also be observed in GBM xenografts with EMP3 depletion. These results would identify EMP3 as a novel therapeutic target that may improve the treatment of CD44-high GBM.

## MATERIALS AND METHODS

### Reagents and cell lines

Antibodies to TGFBR2, cleaved Caspase-3, p-Smad2 (Ser465/467), p-Smad3 (Ser423/425), p-Smad2 (Ser465/467)/p-Smad3 (Ser423/425), Smad2/3, p-NF-κB (Ser536), NF-κB, and CD44 were purchased from Cell Signaling Technology. Antibodies to β-Actin and Ki-67 were from Santa Cruz Biotechnology. Horseradish peroxidase-conjugated goat anti-rabbit or anti-mouse antibodies were purchased from Bio-Rad. TGF-β receptor inhibitor SB431542, IKKβ inhibitor SC-514, TGF-β, and anti-EMP3 antibody (**HPA051163)** were obtained from SIGMA. Plasmid PCMV4-3×HA/IkBα (S32A/S36A) was kindly provided by Addgene plasmid repository. Smad2/3 siRNAs were obtained from Santa Cruz Inc. Human GBM cell lines U251, U373, LN18, A172, and LN229 were obtained from American Type Culture Collection and cultured according to the manufacturer's protocol. Human GBM cell lines U343, and SF295 were kindly provided by the Type Culture Collection of the Chinese Academy of Sciences, Shanghai, China. Normal human astrocytes (Invitrogen) were cultured in GIBCO astrocyte medium (Invitrogen) according to the manufacturer's protocol.

### Lentiviral construction and transduction

Lentiviruses encoding shRNAs silencing EMP3 and control scramble were purchased from Openbiosystem (shRNA sequences are listed in Supplementary Table 1). The production of lentiviral particles and *in* *vitro* infection of cells with the lentivirus was described previously [50].

### Real-time PCR

Total RNA was extracted from GBM cells using TRIzol reagent (Invitrogen) and further purified using the RNeasy kit (QIAGEN). One microgram of total RNA was used to generate cDNA, which was then used for the quantitative PCR using SYBR Green PCR expression assays (Invitrogen). PCR Primers for TGF-β/Smad pathway genes (TGIF, COL6A1, SERPINE1, and TIMP1) are described in Supplementary Table 2.

### Luciferase reporter assay

Analysis on cancer-related pathways activity was performed using the Cignal Finder Pathway Reporter Arrays (SA Biosciences, Fredrick, MD). GBM cells were seeded into a 96-well white plate and incubated overnight at 37°C. Transient transfection was conducted by adding plasmid construct of transcription factor-responsive reporter of each pathway and controls to cells and incubated overnight in a 37°C incubator. Then, cells were further incubated for 48 hours. Each transfection condition was carried in triplicates. After 48 hours, the changes in expression of each pathway in cells with or without EMP3 depletion were determined by measuring the generated firefly and *Renilla* luminescent signals using the Dual-Glo Luciferase Assay system (Promega, Madison, WI) on the Glomax machine (Promega, USA). The relative luciferase units were determined by dividing the firefly to *Renilla* luciferase activity ratio. The luciferase activity of scramble shRNA control was regarded as 100%.

### Soft agar assay

Anchorage-independent growth of glioma cells was tested as described previously [50]. The 2 mL culture medium with 0.5% agar was first plated into each well of a 6 cm culture dish. After the agar solidified, each well received another 2 mL of 0.35% agar in culture medium containing 1×10^4^ cells. After 4 weeks, colonies were fixed and stained with 0.1% crystal violet. The number of colonies was determined microscopically by manually counting from triplicate wells for each cell line.

### Western blot analysis

Cell lysates were prepared with cell lysis buffer containing 1% NP-40, 0.5% sodium deoxycholate, 0.1% SDS, and protease inhibitor cocktail. Western blotting analysis was conducted as described previously [51]. After standard SDS-PAGE and western blotting procedures, proteins were visualized using the ECL system (Amersham Biosciences).

### Immunofluorescence staining

Cells were cultured on glass coverslips in six-well plates, rinsed thrice with PBS, fixed with 3.7% paraformaldehyde for 15 minutes, and blocked with 5% normal goat serum for 1 hour. The cells were immunostained by using primary antibodies specific to various antigens. Goat anti-mouse IgG Alexa 488 or goat anti-rabbit IgG Alexa 546 was used as the secondary antibody. Images were taken under a Zeiss axiocam fluorescence microscope using Axiovision software (Zeiss, Germany).

### TUNEL assay

The level of apoptosis in GBM cells was assessed using the terminal deoxynucleotidyl transferase-mediated deoxyuridine triphosphate nick end-labeling (TUNEL) method [50]. The percentage of TUNEL- labeled cells in each section was determined at a magnification of 400× by counting 500 cells in five randomly selected fields.

### Cell proliferation assay

To determine the effect of EMP3 silencing on cell proliferation, GBM cells were infected with scramble or shRNA-targeting lentivirus for 48 hours, dissociated into single cell, plated onto 6-well plates (2×10^4^ cells/well), and incubated for 5 days. The cultures were trypsinized, and the number of viable cells in each group was counted with a hemocytometer using 0.2% trypan blue exclusion.

### Immunohistochemistry (IHC)

IHC was performed on seventy formalin-fixed, paraffin-embedded primary GBM tissue sections (*n* = 60). These patients were performed neurosurgery and diagnosed with primary GBM (2012-2013) in Xiangya hospital (Central South University). The protocols were approved by the research ethics committee in Central South University with informed consent having been obtained from all patients. Antigen-antibody reactions were detected by exposure to 3,3-diaminobenzidine and hydrogen peroxide chromogen substrate (DAKO, Denmark). Slides were counterstained with hematoxylin and mounted. The negative controls were incubated with non-immune mouse IgG. IHC staining was scored on a 0-to-4 scale according to the staining intensity and extent as described previously [52]. Briefly, 0, negative or weak staining in 10% or less of cells; 1, weak staining in 11%-30% of cells; 2, weak staining in more than 30% of cells or moderate staining in less than 30% of cells; 3, moderate staining in 30%-60% of cells; and 4, moderate or strong staining in more than 60% of cells. The IHC results were independently reviewed by 2 pathologists (J.H and S.W). An average score was assigned for GBM section based on the staining positivity of four randomly selected fields in each section. The score ( ≤ 2) or ( > 2 ) was regarded as low expression or high expression, respectively.

### Animal studies

Immunocompromised nude mice were obtained from the breeding facility at the animal center of Central South University. All animal studies were performed in accordance with institutional ethical guidelines for experimental animal care. For subcutaneous xenograft study, 2×10^6^ LN18 cells were inoculated into the flank of nude mice (*n* = 6). In order to determine tumor volume by external caliper, the greatest longitudinal diameter (a) and the greatest transverse diameter (b) were determined. Tumor volume based on caliper measurements was calculated by the modified ellipsoidal formula: tumor volume (mm3) = a×b^2^/2. For survival analysis, 2 ×10^5^ LN18 cells were injected stereotactically into 4-week-old nude mice cortex, following administration of general anesthesia. The injection coordinates were 3 mm to the left of the midline, 2 mm anterior to the lambdoid suture, and 3 mm deep. The incision was closed with wound clips and removed 4 days after inoculation. Animals that died, lost weight, or developed neurological deficits within 24 hours of cell injection were excluded. The animals were monitored daily until signs of neurological deficit developed, at which time they were euthanized and their brains removed. For histopathological analysis, the mouse brain xenografts embedded in optimum cutting temperature (OCT) were stored in liquid nitrogen overnight, and then sectioned at 5 mm thickness on a MicromHM200 cryotome (Eryostar). Hematoxylin and eosin (H&E) stained sections were evaluated for evidence of tumor.

### The cancer genome atlas data analysis

Array mRNA data (Affymatrix U133 2.0) from GBM patients were downloaded from the TCGA project data portal (http://cancergenome.nih.gov/dataportal). Details on the data processing and platforms are in the publication describing the GBM data analysis [23].

### Statistical analysis

Statistical evaluations were carried out using SPSS 10.0 software (SPSS Inc, U.S.A). Error bars throughout the figures indicate S.D. Student's *t* test was used to compare means of two groups. O*ne*-*way* analysis of variance (*One*-*Way ANOVA*) was used to compare means of three or more groups. Kaplan-Meier curves of overall survival were drawn and survival in the groups was compared using the log-rank test. For all tests, the level of statistical significance was set at *P* < 0.05. All statistical tests were two-sided.

## SUPPLEMENTARY MATERIALS FIGURES AND TABLES


