# Supplementary material for: Epithelial membrane protein 3 regulates TGF-β signaling activation in CD44-high glioblastoma
Source: Oncotarget. 2016 Aug 5;8(9):14343–58. doi: 10.18632/oncotarget.11102 (PMC5362410; doi:10.18632/oncotarget.11102)
Supplement: Supplementary file 1 [file oncotarget-08-14343-s001.pdf]

Epithelial membrane protein 3 regulates TGF-β signaling activation in CD44-high glioblastoma

Supplementary Material

Supplementary Tables

Table 1: shRNA oligonucleotide sequence

Scramble shRNA:
5 -GTCACGATAAGACAATGAT-3

EMP3 shRNA-1:
5 -TGAATGGCATAGATCAAGGCG-3

EMP3 shRNA-2:
5 -ATTCTCGCTGACATTACTGCA-3

Table 2: Real-time PCR Primers for SERPINE1, TIMP1, TGIF, and COL6A1

SERPINE1:
Forward: GAGGTGCCTCTCTCTGCCCTCACCAACATT
Reverse: AGCCTGAAACTGTCTGAACATGTCG

TIMP1:
Forward: CTTCTGGCATCCTGTTGTTG
Reverse: AGAAGGCCGTCTGTGGGT

TGIF:
Forward: TGGCAGTGAGACTGAGGATG
Reverse: TGGATCTTTGCCATCCTTTC

COL6A1:
Forward: CTGGGCGTCAAAGTCTTCTC
Reverse: ATTCGAAGGAGCAGCACACT

Supplementary Figures

Figure S1

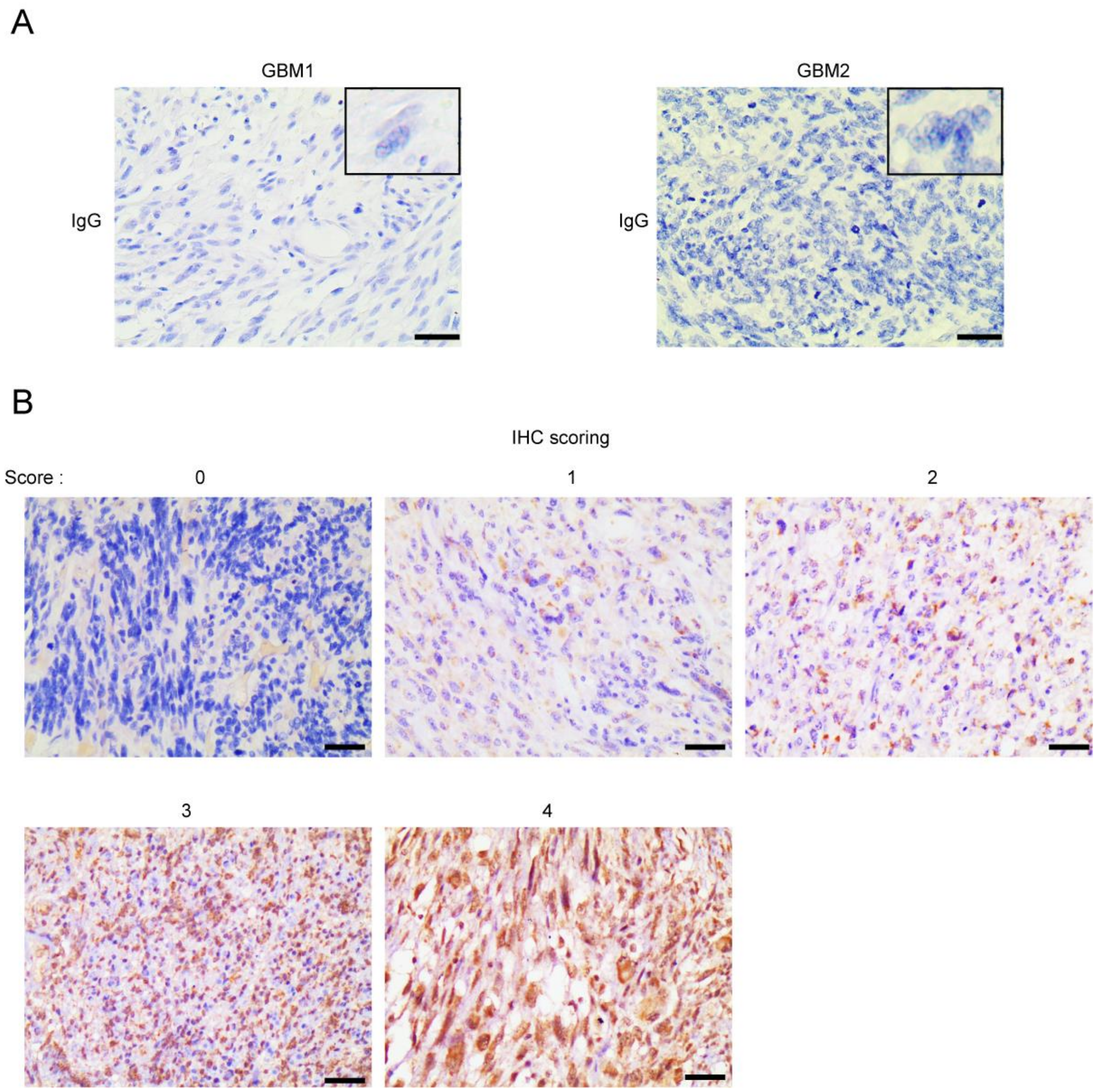

Figure S1. A. Isotype IgG staining on GBM1 and GBM2 tissue sections. Bars: 100μm. B. IHC scoring on EMP3 staining in GBM sections.

Figure S2

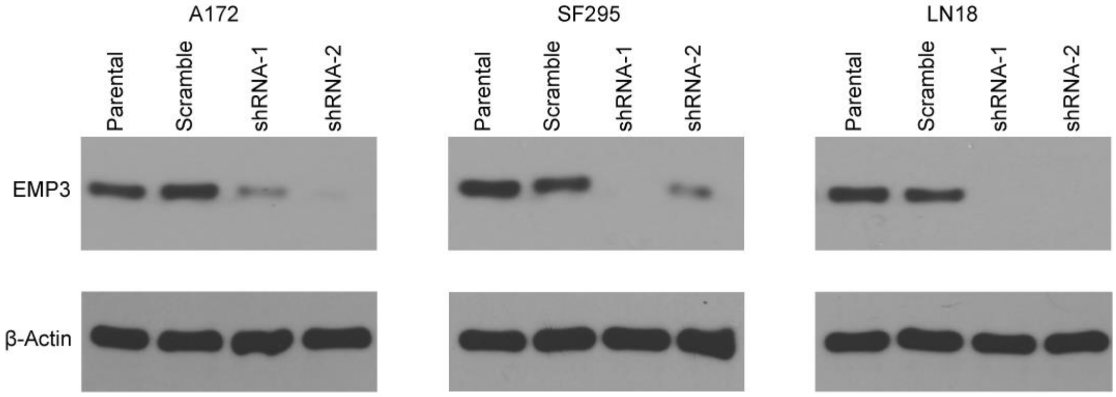

Figure S2: Silencing effect of EMP3 shRNAs in GBM cell lines.

Figure S3

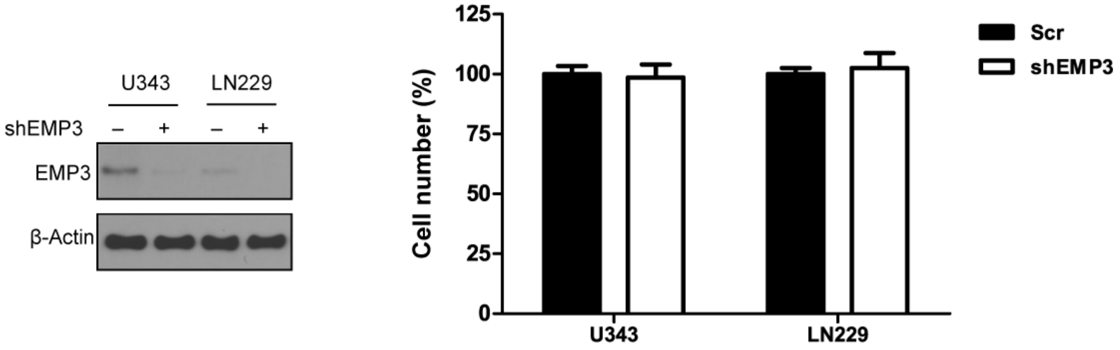

Figure S3: Knockdown of EMP3 did not affect the cell viability in EMP3-low GBM cell lines.

Figure S4

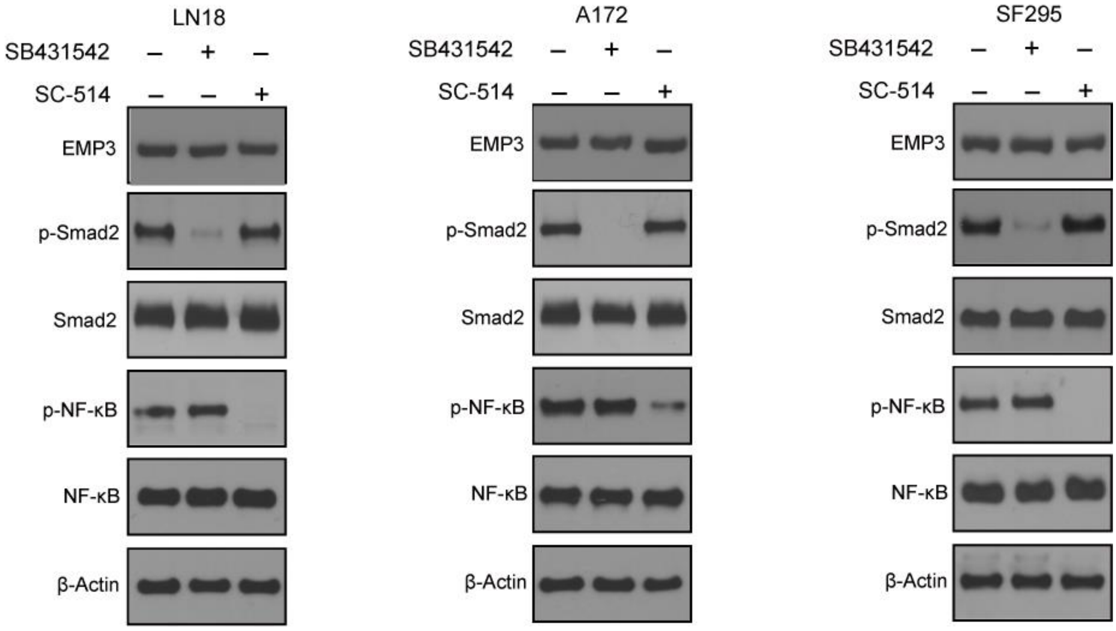

Figure S4. Effects of TGF-β receptor inhibitor SB431542 and IKKβ inhibitor on GBM cell lines

Figure S5.

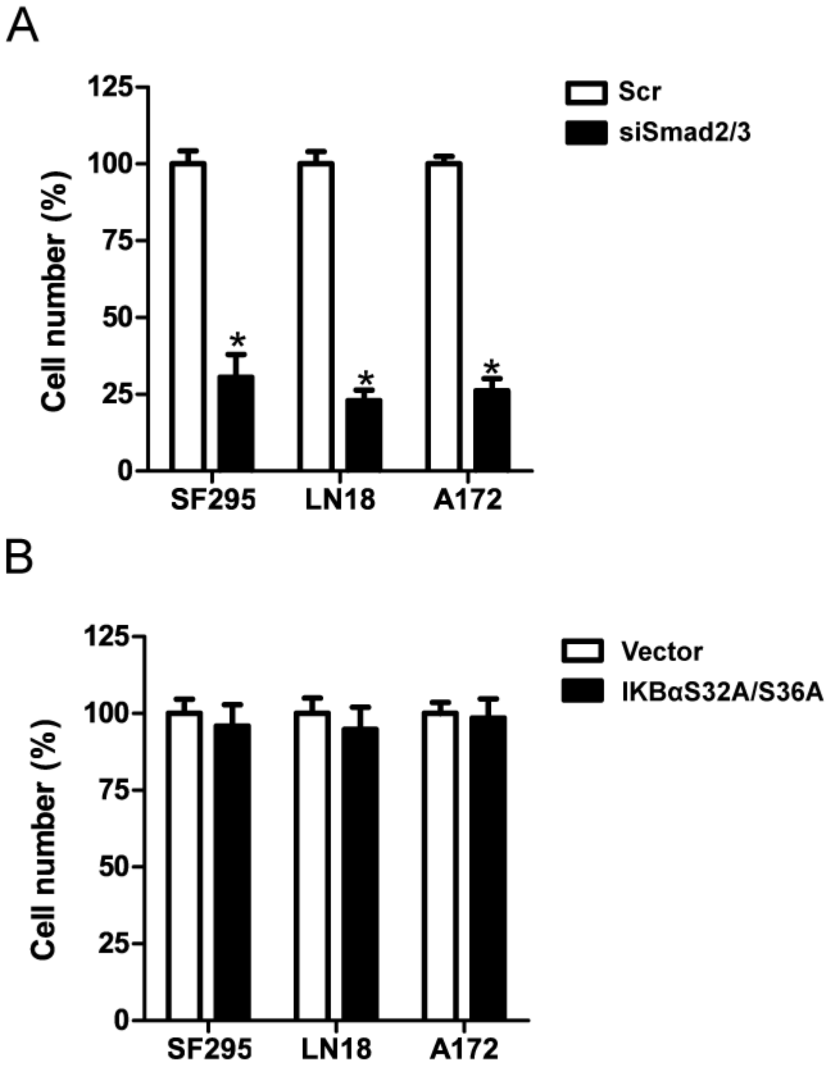

Figure S5. Effect of Smad2/3 silencing or over-expression of IKBαS32A/S36A on cell proliferation in GBM cell lines.
